# New Chelate Resins Prepared with Direct Red 23 for Cd^2+^, Ni^2+^, Cu^2+^ and Pb^2+^ Removal

**DOI:** 10.3390/polym14245523

**Published:** 2022-12-16

**Authors:** Nicoleta Mirela Marin, Anton Ficai, Lucian Alexandru Constantin, Ludmila Motelica, Roxana Trusca

**Affiliations:** 1 National Research and Development Institute for Industrial Ecology ECOIND, Street Podu Dambovitei no. 57-73, District 6, 060652 Bucharest, Romania; 2 Science and Engineering of Oxide Materials and Nanomaterials, Faculty of Chemical Engineering and Biotechnologies, University Politehnica of Bucharest, Gh. Polizu 1-7, 011061 Bucharest, Romania; 3 National Center for Micro and Nanomaterials, University Politehnica of Bucharest, 011061 Bucharest, Romania; 4 Academy of Romanian Scientists, Ilfov Street 3, 050044 Bucharest, Romania

**Keywords:** Amberlite XAD7HP, Amberlite IRA 402, ion exchange mechanism, π-π interactions, chelation, simultaneous enrichment, higher selectivity, cadmium, nickel, copper, lead

## Abstract

In this paper, two chelate resins prepared by a simple procedure were used for the removal of Cd^2+^, Ni^2+^, Cu^2+^, and Pb^2+^ (M^2+^) from aqueous solutions. Amberlite IRA 402 strongly basic anion exchange resin in Cl^−^ form (IRA 402 (Cl^−^) together with Amberlite XAD7HP acrylic ester co-polymer (XAD7HP) were functionalized with chelating agent Direct red 23 (DR 23). The chelate resins (IRA 402-DR 23 and XAD7HP-DR 23) were obtained in batch mode. The influence of interaction time, pH and the initial concentration of DR 23 solution was investigated using UV-Vis spectrometry. The time necessary to reach equilibrium was 90 min for both resins. A negligible effect of adsorption capacity (*Q_e_*) was obtained when the DR 23 solution was adjusted at a pH of 2 and 7.9. The *Q_e_* of the XAD7HP resin (27 mg DR 23/g) is greater than for IRA 402 (Cl^−^) (21 mg DR 23/g). The efficiency of chelating resins was checked via M^2+^ removal determined by the atomic adsorption spectrometry method (AAS). The M^2+^ removal by the IRA 402-DR 23 and XAD7HP-DR 23 showed that the latter is more efficient for this propose. As a consequence, for divalent ions, the chelated resins followed the selectivity sequence: Cd^2+^ > Cu^2+^ > Ni^2+^ > Pb^2+^. Additionally, Cd^2+^, Cu^2+^ and Ni^2+^ removal was fitted very well with the Freundlich model in terms of height correlation coefficient (R^2^), while Pb^2+^ was best fitted with Langmuir model for IRA 402-DR 23, the Cu^2+^ removal is described by the Langmuir model, and Cd^2+^, Ni^2+^ and Pb^2+^ removal was found to be in concordance with the Freundlich model for XAD7HP-DR 23. The M^2+^ elution from the chelate resins was carried out using 2 M HCl. The greater M^2+^ recovery from chelating resins mass confirmed their sustainability. The chelate resins used before and after M^2+^ removal by Fourier transform infrared spectroscopy (FTIR) and scanning electron microscopy (SEM) analysis were evaluated.

## 1. Introduction

Heavy metals, including copper, cadmium, lead, nickel, arsenic, chromium, etc., are non-biodegradable and tend to accumulate in the environment [1,2,3]. Water pollution with heavy metals resulting from anthropogenic activities is one of the global risks to humans [4]. At the same time, serious pollution of ecosystems with heavy metals can be observed with the increase in industrialization [5]. Over time, non-conventional and conventional materials were also involved in the removal of metal ions [6,7,8]. Nowadays, the feasibility of different chemically modified polymers, namely chelating polymers, was tested in the field of wastewater treatment in search of more effective means of depollution. Furthermore, to improve their adsorption capacity, chemical modifications can be performed with chelating ligands that have the following functional groups in their structure: –COO^–^, –N=N–, –CO^–^, –SO_3_^–^, –NH_2_, –OH and –SH [9,10,11]. Thus, the recyclable aims including regeneration and reuse of chelating polymers are important attributes of green technologies. In recent years, chelating resin, i.e., 1,8-(3,6-dithiaoctyl)-4-polyvinylbenzenesulphonate, was evaluated for Pb^2+^ and Cd^2+^ removal from aqueous matrices. The behavior of chelating resin regarding interactions between chelating agent and metal ions has been monitored. The sorption efficiency was examined by batch, and column methods also showed a sorption over 99.0% for both metal ions [12]. Additionally, m-Phenylenediamine-Amberlite XAD-4 resin was evaluated for Pb^2+^ and Cd^2+^ removal. The concentrations studied were performed in the range of 5–100 mg Pb^2+^ and Cd^2+^, at 30 min stirred time and 0.2 g chelating resin. It was found that for 20, 60, 80 and 100 mg/L, respectively, chelating resin removed all Pb^2+^ and Cd^2+^ from supernatant solutions [13]. Cai et al. tested a porous impregnated resin, i.e., pillar [5] arene-based diglycolamide onto Amberlite XAD-7, and the obtained resin was used for lanthanides recovery. Thus, significant environmental protection and the ecofriendly recovery of resources were obtained when the impregnated resin is used for depolluting simulated nuclear wastewater. Experimental data were fitted, and adsorption capacity for Eu^3+^ was 33.3 mg/g [14]. In another study, Amberlite XAD-7HP resin was impregnated with Cyanex 923 for Cr^6+^ removed from acidic media. The adsorption mechanism was evaluated before and after impregnation. The adsorption capacity was 28.2 mg Cr^6+^/g impregnated resin compared with 16.9 mg Cr^6+^/g resin. Additionally, Cr^6+^ was released efficiently from impregnated XAD-7HP resin by NaOH 0.1 M obtaining a quantitative elution that reached 92% for the first stage [15]. Additionally, two environmentally ecofriendly adsorbents Amberlite IRA-93 (A-IRA-93) and its modified form (mA-IRA-93) obtained with methylene phosphoric acid (DMPA) for uranyl ions extraction were used. The adsorption capacity was 8.34 mg UO_2_^2+^/g for mA-IRA-93 and 5.60 mg UO_2_^2+^/g for A-IRA-93 [16]. Among the chelating resins tested, Amberlite XAD7HP had the most efficient results. XAD-7HP resin was impregnated with PC-88A and Versatic 10. The applicability of impregnated resin recovery Sc^3+^ in the presence of Al^3+^, Fe^3+^, Zr^4+^, Mn^2+^, Co^2+^, Cu^2+^, Ni^2+^, and Zn^2+^. Forty-eight milligrams of Sc^3+^/g resin was the maximum loading capacity of impregnated resin [17].

In the present study, the possible chelating mechanism is inferred between two–N=N– groups, sulfonic groups (–SO_3_^–^) that can be free after adsorption of DR 23 and diffusion into porous structure, and hydrogen bonding and van der Vaal forces can be considered. In this study, a new type of chelating resin was obtained by adsorption of DR 23 onto IRA 402 and XAD7HP. In this study, the chelating procedures were evaluated by considering the following parameters: (i) the influence of interaction time between DR 23 and resins; (ii) the influence of pH on the DR 23 adsorption into resins masses; (iii) the initial concentration of DR 23; (iv) the stability of chelating resin; (v) M^2+^ adsorption/desorption; (vi) kinetics and isotherms studies, which were evaluated to understand the M^2+^ adsorption; (vii) the M^2+^ interaction with chelating resins using FTIR and SEM techniques performed. To our knowledge, for the first time, this new chelating resin has been applied for Cd^2+^, Ni^2+^, Cu^2+^ and Pb^2+^ removal from acid aqueous matrices.

## 2. Materials and Methods

### 2.1. Chemicals

To carry out the experimental part of this paper, the following chemicals were used: DR 23 (7-[[6-[(4-acetamidophenyl)diazenyl]-5-hydroxy-7-sulfonatonaphthalen-2-yl] carbamoylamino]-4-hydroxy-3-phenyldiazenylnaphthalene-2-sulfonate) 30% dye content, strongly basic anion exchange resin Amberlite IRA 402, 50 mesh, in Cl^−^ form, and Amberlite XAD7HP 20–60 mesh were purchased from Sigma Aldrich. CRM solutions of 1000 mg/L Ni(NO_3_)_2_, Cd(NO_3_)_2_, Pb(NO_3_)_2_ and Cu(NO_3_)_2_ and 37 % HCl and 50% NaOH (Merck) were used for testing the chelating resins. Methanol with purity ≥ 99.9% for HPLC (MeOH) Honeywell and ethanol (EtOH) (Merck) were used in regeneration studies of the chelating resins.

### 2.2. Equipment

For the spectrometric analysis, a DR/5000 TM spectrometer (Hach Lange, Düsseldorf, Germany) was used. The Perkin Elmer PinAcle 900T (Perkin Elmer, Norwalk, CT, USA) atomic absorption spectrometer was used for the quantitative determination of M^2+^. The pH of the supernatant solutions was checked using the pH meter HI 255 Hanna Instruments, Nijverheidslaan, Belgium. For obtained chelating IRA 402-DR 23 and XAD7HP-DR 23 resins, as well as for the adsorption/desorption of M^2+^ on the chelating resins, a horizontal mechanical stirrer GFL 3017 (Bremen, Germany) was used. Infrared spectra of both chelating resins were recorded by a Nicolet iS50FT-IR (Thermo Fisher Scientific, Waltham, MA, USA). The Quanta Inspect F50 electron microscope (Eindhoven, The Netherlands) was used for examining the change induced by the presence of metal ions from the solution on the beads’ surfaces.

### 2.3. Preparation of Working Solution

The stock solution of 700 mg/L was obtained by weighing an appropriate amount of DR 23 and dissolving it in water, and subsequently, the obtained solution was transferred to a 500 mL volumetric flask. Aqueous solutions by different concentrations of HCl were obtained by dilution from the concentrated solution of 37% HCl. Additionally, 2 M NaOH solution was obtained by dilution from the 50% NaOH solution.

### 2.4. Analytical Method Used for DR 23 and M^2+^ Determination

#### 2.4.1. UV-Vis Determination of DR 23

**Figure 1:**
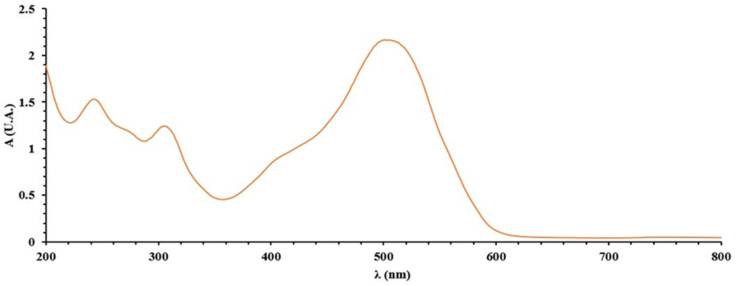
Representation of UV-Vis spectra for 60 mg/L DR 23.

Beer’s law was verified in the 50–100 mg/L range for the spectrometric determination of the chelating agent (DR 23). For this purpose, accurately measured volumes of DR 23 from the stock solution were added to 25 mL volumetric flasks. The flasks were filled to the mark with ultrapure water and the absorbance of the solution (A) toward the blank sample (ultrapure water) was recorded. From Figure 1, the UV-Vis spectrum of DR 23 was read to set the optimum maximum wavelengths. The DR 23 spectrum has three maximum wavelengths at 507, 307 and 243 nm.

To draw the calibration line, as well as for the chelating studies, a fixed wavelength (λ) at 507 nm was chosen. The calibration curve A = 0.0410 C − 0.2690 was used for the quantitative determination of DR 23, where C (mg/L) is the concentration of DR 23 in the supernatant solution. Additionally, the limit of determination (LD) was 0.73 mg/L DR 23 (corresponding to ten times of standard deviation of the blank solution measured at 507 nm) [18].

#### 2.4.2. AAS Method for M^2+^ Determination

M^2+^ from supernatant obtained between and after the interaction with chelating resin by AAS was evaluated. The calibration curves in the 0.1–0.5 mg/L range were studied corresponding to each M^2+^. Thus, from 1000 mg/L stock solutions of CRM, the Ni(NO_3_)_2_, Cd(NO_3_)_2_, Pb(NO_3_)_2_ and Cu(NO_3_)_2_ the calibration curves were prepared in 50 mL volumetric flasks by dilution, applying the law of normality. Each standard solution was read, and A = f (M^2+^ concentration) was recorded. The calibration curves obtained—y = 0.0507x − 0.0001 for Ni^2^, y = 0.0924x + 0.0006 for Cu^2+^, y = 0.0102x + 0.0009 for Pb^2+^ and y = 1.3905x + 0.0383 for Cd^2+^—were used to determine the M^2+^ in the adsorption/desorption studies for testing the chelated resins obtained.

Additionally, LDs of M^2+^ were obtained as being ten times the standard deviations to the blank solution read at the specific wavelength (232 nm for Ni^2+^, 324.75 nm Cu^2+^, 283.31 nm for Pb^2+^, and 228.8 nm for Cd^2+^) and were determined as being 0.0027 mg/L Ni^2+^, 0.0035 mg/L Cu^2+^, 0.0040 mg/L Pb^2+^, and 0.0031 mg/L Cd ^2+^.

### 2.5. Procedure for Determination of the Interaction Time for Optimal Adsorption of DR 23 in IRA 402 (Cl^−^) and XAD7HP Resins

First, 0.5 g of IRA 402 (Cl^−^) and XAD7HP resins were weighed in Erlenmeyer flasks. Then, 0.05 L of 300 mg/L DR 23 solution was added to the weighed resins. The obtained mixtures were stirred at the following interaction times of 15, 30, 45, 60, 75, 90, and 105 min, respectively, and stirred at 175 rpm (25 ± 2 °C). After stirring, IRA 402 (Cl^−^) and XAD7HP loaded with DR 23 were filtered on filter paper, and the amount of DR 23 retained in the resin mass was determined from the filtered solutions using the calibration curve presented in Section 2.4. The adsorption capacity evaluated as a function of interaction time (*Q_t_* (mg/g)) was calculated with the following equation [19,20,21,22]:

(1) $$Q_{t\;}=\frac{\left(C_{i}-C_{t}\right)V}{m}$$

where *C_i_* and *C_t_* (mg/L) are the initial concentration of DR 23 and at the time (*t*) min, *m*(g) is the mass of the dry IRA 402 and XAD7HP, and *V*(L) is the volume of the chelating agent (DR 23). All experimental data were collected in duplicate, and the average data were applied for *Q_t_* determination.

### 2.6. Procedure for Studies the Influence of pH

Over 0.5 g of IRA 402 resin (Cl^−^) and XAD7HP resins, 0.05 L of 200 mg/L DR 23 solution was added, the pH was adjusted to 2 in the presence of 10^−4^ M HCl. The obtained mixtures were stirred at 175 rpm for 90 min at 25 ± 2 °C. At the end of stirring, the mixtures were filtered, and the quantity of DR 23 that was not retained in the resins’ mass was determined spectrometrically. For the solution of DR 23 with pH = 7.9, the same experimental procedure was applied. For the adsorption capacity obtained at equilibrium (*Q_e_* (mg/g) for DR 23 on IRA 402 and XAD7HP mass (m(g)), the following equation was applied [23]:

(2) $$Q_{e\;}=\frac{\left(C_{i}-C_{e}\right)V}{m}$$

where *C_e_* (mg/L) is the DR 23 concentration at equilibrium. All experimental data were collected in duplicate and the average data were applied for *Q_e_* determination.

### 2.7. Procedure for Obtained Chelate IRA 402 (Cl^−^) Resin

First, 0.5 g of dry resin IRA 402 (Cl^−^) and 0.05 L of DR 23 at different concentrations—50, 100, 150, 200, 250, 300 and 350 mg/g—were introduced into 250 mL Erlenmeyer flasks. The obtained mixtures were stirred for 90 min at 175 rpm at 25 ± 2 °C. At the end of the 90 min, the chelate resin IRA 402-DR 23 was filtered and the amount of DR 23 that was not retained in the resin was determined spectrometrically based on the calibration curve presented in Section 2.4. All experimental data were collected in duplicate, and the average data were applied for *Q_e_* determination.

### 2.8. Procedure for Obtained Chelate Amberlite XAD7HP Resin

The preparation of the resin with chelating properties, XAD7HP-DR 23, started from the conventional resin of poly-acrylic acid esters XAD7HP. For this purpose, 0.5 g of dry resin XAD7HP and 0.05 L of DR 23 at different concentrations—50, 100, 150, 200, 250, 300 and 350 mg/g—were introduced into 250 mL Erlenmeyer flasks. The obtained mixtures were stirred for 90 min at 175 rpm (25 ± 2 °C). At the end of the 90 min, the chelate resin XAD7HP-DR 23 was filtered, and the amount of DR 23 that was not retained in the resin was determined spectrometrically using the calibration curve presented in Section 2.4. All experimental data were collected in duplicate, and the average data were applied for *Q_e_* determination.

### 2.9. Procedure for Evaluate the Stability of Chelate Resins

The stability of IRA 402-DR 23 (21.2 mg DR 23/g) and XAD7HP-DR 23 (27.2 mg DR 23/g) resins in the presence of the desorption agents 2 M HCl, 2 M NaOH, MeOH and EtOH was evaluated. Chelating resins were weighed in Erlenmeyer flasks and treated with 0.05 L of 2 M HCl, 2 M NaOH, MeOH and EtOH. The obtained mixtures were stirred at 175 rpm for 30 min at 25 ± 2 °C. At the end of the stirring time, the IRA 402-DR 23 and XAD7HP-DR 23 resins were filtered on filter paper, and the effluent solutions were analyzed spectrometrically. The amount of DR 23 released in the effluent solutions was determined using the calibration curve presented in Section 2.4. Additionally, the DR 23 desorption (*D* (%)) was evaluated using the following equation [24]:

(3) $$D\;\left(\%\right)\;=\frac{A}{\;B}\times 100$$

where *A* is the DR 23 amount (mg) released in supernatant after desorption studies and *B* is the DR 23 amount (mg) that remains in the chelating resins’ mass after desorption studies. All experimental data were collected in duplicate, and the average data were applied for *D* (%) determination.

### 2.10. Procedure for Studies on the Influence of Contact Time between Cd^2+^, Ni^2+^, Cu^2+^ and Pb^2+^ and Chelate Resins

To study the influence of the contact time for 0.5 g of chelated resin (IRA 402-DR 23 and XAD7HP-DR 23), 0.05 L of 0.6 mg/L M^2+^ concentration was added to each sample. The obtained mixtures were subjected to 175 rpm (25 ± 2 °C) mechanical stirring at specified time intervals, which were 15, 30, 45, 60, 75, 90 and 120 min. After the end of each studied time interval, the concentration of M^2+^ was determined from the supernatant solution. The optimal contact time was established when the concentration of M^2+^ in the supernatant solutions remained constant. The adsorption capacity of chelate resin as a function of interaction time (*Q_t_* (mg/g)) for M^2+^ removal was calculated with Equation (1). All experimental data were collected in duplicate, and the average data were applied for *Q_t_* determination.

### 2.11. Procedure for Cd^2+^, Ni^2+^, Cu^2+^ and Pb^2+^ Removal Using Chelate Resins

For Cd^2+^, Ni^2+^, Cu^2+^ and Pb^2+^ removal by chelate resins, approximately 0.5 g of IRA 402-DR 23 (21.2 mg DR 23/g) and XAD7HP-DR 23 (27.2 mg DR 23/g) resins were stirred with 0.05 L solutions of M^2+^ (pH = 2.5), which varied from 0.05 mg/L (S1) to 0.1 mg/L (S2), 0.15 mg/L (S3), 0.2 mg/L (S4), 0.3 mg/L (S5), 0.4 mg/L (S6) and 0.6 mg/L (S7), were stirred for 60 min at 175 rpm (25 ± 2 °C). After stirring, IRA 402-DR 23-M^2+^ (S1 to S7) and XAD7HP-DR 23-M^X+^ (S1 to S7) were filtered, and the concentration of each M^2+^ cation was determined by AAS using the calibration curves presented in Section 2.4. The M^2+^ removal was determined by considering the difference between the initial M^2+^ concentration from the solution (*C_i_* (mg/L)) and the final concentration in the eluent (*C_e_* (mg/g)) after the adsorption process. The following equation was applied to calculate M^2+^ efficiency removal *R* (%) [24].

(4) $$R\;\left(\%\right)=\frac{C_{i}-C_{e}}{C_{i}}\times 100$$

All experimental data were collected in duplicate, and the average data were applied for *R* (%) determination.

### 2.12. Procedure for M^2+^ Recovery from IRA 402-DR 23-M^2+^ and XAD7HP-DR 23-M^2+^

IRA 402-DR 23-M^2+^ (S1 to S7) and XAD7HP-DR 23-M^2+^ (S1 to S7) solid phases obtained in Section 2.11 (0.5 g) were stirred for 60 min with 0.05 L 2 M HCl in 250 mL Erlenmeyer flask for 60 min at 175 rpm (25 ± 2 °C). After stirring, the resins were filtered, and the filtrated solutions were analyzed by AAS using calibration curves presented in Section 2.4. All experimental data were collected in duplicate and the average data were applied for *D* (%) determination.

## 3. Results and Discussions

### 3.1. Effect of Interaction Time (DR 23-Resin)

**Figure 2:**
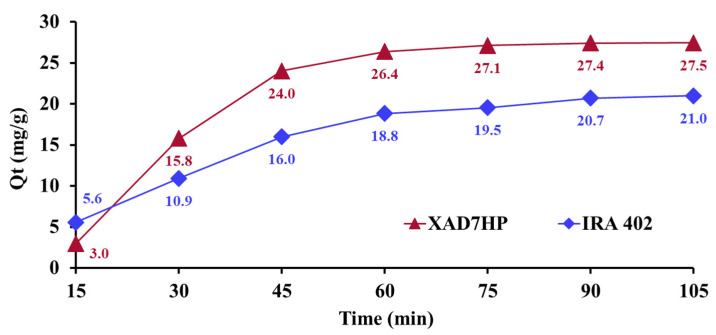
Influence of interaction time on adsorption of DR 23 on IRA 402 (Cl^−^) and XAD7HP.

The effect of interaction time regarding the DR 23 adsorption onto IRA 402 (Cl^−^) and XAD7HP mass is presented in Figure 2. The quantity of the chelate agent adsorption onto IRA 402 (Cl^−^) and XAD7HP increased with the increase in the interaction time from 15 to 90 min. In the first 30 min, more than half of the chelating agent was retained on the resins’ mass. It is observed that the time required to obtain chelate resins is 90 min when Q_t_ varies insignificantly. Marin et al. studied the effect of interaction time between the chelating agent (Acid Blue 113 (AB 113)) and the strongly basic anion exchanger resin IRA 402 in Cl^−^ form in the range of 30 to 135 min. It was observed that the equilibrium was influenced by the interaction time, such that in the first step (30–90 min), the AB 113 adsorption was fast. Subsequently, in the second step, Q_t_ values varied insignificantly and equilibrium was reached [25]. Additionally, XAD7HP resin was impregnated with procainamide (PHA) and lidocaine (LID), and the influence of the interaction time was evaluated within the 30 to 80 min range. The time necessary to reach equilibrium for impregnation of XAD7HP was determined at 60 min for both pharmaceutical compounds [19].

### 3.2. The Influence of pH

pH is one of the parameters that may influence the chelating efficiency. For this reason, the adsorption process of DR 23 at pH = 2 and 7.9 on IRA 402 and XAD7HP was studied. When experimental studies were carried out, the maximum adsorption of DR 23 for anion exchange resin IRA 402 (Cl^−^) was obtained at pH = 7.9 with a value of Q_e_ of 15.1 mg/g, while at pH = 2, the Q_e_ is only 14.2 mg/g.

The insignificant behavior at pH 2 can be explained as follows: the height structure of DR 23 can restrict the ion exchange mechanism that is favored by the sulfonic groups of DR 23 and the functional groups of the strongly basic anionic resin in the Cl^−^ form (R-N^+^(CH_3_)_3_Cl^−^).

Therefore, for the obtained chelate IRA 402 (Cl^−^) resin, we can provide the following hypothesis: besides the ion exchange adsorption, the aromatic structure of DR 23 favors also its adsorption on the styrene divinylbenzene resin by physical adsorption.

Additionally, the adsorption of DR 23 by the XAD7HP is achieved through physical adsorption and through the molecular sieve effect that the acrylic support exhibits on the DR 23 molecules with which it comes into interaction.

For Amberlite XAD7HP resin, a better adsorption was obtained at pH = 2, and Q_e_ is 18.7 mg/g. While at pH = 7.9, Q_e_ was 17.0 mg/g.

Taking into account the small influence of the pH of DR 23 solutions on the adsorption capacity for IRA 402 (Cl^−^) and XAD7HP, future experimental studies were evaluated at pH = 7.9, which is the pH of the solution. Marin et al. studied the influence of pH when strongly basic anion exchange resin (IRA 400) was used for Acid Orange 10 (AOG 10) removal. AOG 10 was used in disodium salt that was dissociated in water and exists in two complete negative forms. In addition, at a pH higher than 9, the phenolic group is ionized. In such conditions, all three anion groups may be involved at the ion exchange equilibrium and the influence of AOG 10 adsorption onto resin mass was studied. For all pH studies that were evaluated at different pH values from 1.02 up to 10.3, the adsorption of AOG 10 was not suggested, and the percent removal varied from 99.9% to 99.7% [26]. A similar result was presented by Anjali et al., where Amberlite XAD-7HP resin (X7), was impregnated (IMPXD7) with Aliquat 336 (A 336) and used for the removal of Reactive Blue-13 (RB-13) using various pH ranges. For this reason, batch experiments were performed using 4 g/L IMPXD7 and 50 mg/L RB-13 for 1 h in the pH range between 2.18 and 11.82. The obtained results showed insignificant percentage removal that varied from 98.76 up to 95.03 when the pH of the supernatant was evaluated from acidic to alkali medium. Efficient removal ~99% at pH = 5.75 was obtained. This behavior can be quantified by an adsorbent point of zero/neutral charge that is 6.88. At pH < pH_o_, the surface of the adsorbent is positively charged. Therefore, in an acidic environment, the retention rate of the RB-13 dye increases. Thus, the interaction between the anionic groups of the RB-13 dye and the positive groups on the surface of the adsorbent improves the adsorption of the dye. At the same time, when pH > pH_o_, the surface of the adsorbent is probably negatively charged, resulting in a decrease in dye adsorption. It was observed that in both acidic and alkaline media, no changes in the adsorption efficiency of the RB-13 dye were highlighted. Considering these observations, experimental studies were carried out at the pH of the dye solution [27].

### 3.3. Influence of Chelate Agent Concentration

The chelating resins were achieved by the batch method. Additionally, the functionalization was carried out by taking into account the exchange capacity of the resin presented in the datasheet of the IRA 402 (Cl^−^) resin. The ion exchange capacity determined theoretically was 0.9765 g DR 23/0.8 g IRA 402 dry resin [28]. At the same time, for the XAD7HP resin, functionalization was achieved by gradually increasing the concentration until saturation was obtained.

**Figure 3:**
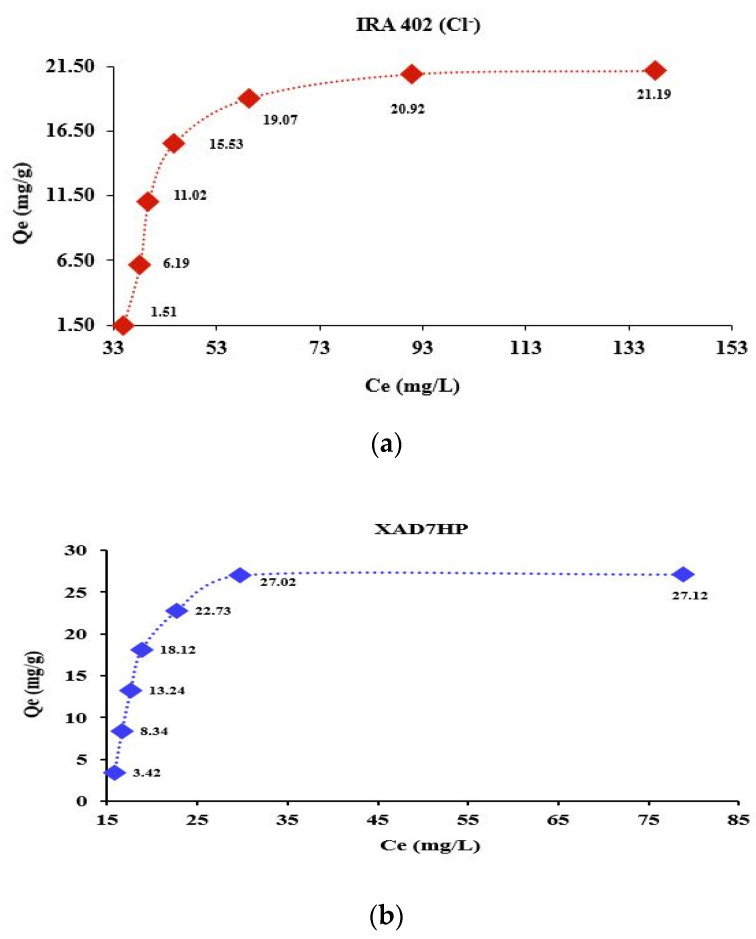
Influence of DR 23 initial concentration for (**a**) IRA 402 (Cl^−^) and (**b**) XAD7HP.

Thus, taking into account the previously stated aspects, the range of concentrations varied from 50 to 350 mg/L DR 23. Figure 3a,b show the effect of the initial concentration on the resin mass IRA 402 (Cl^−^) and XAD7HP, respectively.

Significant adsorption on the resin mass was obtained at high concentrations of DR 23. As one can see, the resin mass may have retained only a limited amount of DR 23 later; even if the concentration of DR 23 is increased, significant adsorption is no longer observed (Figure 3a,b). Additionally, the *Qe* values determined for the XAD7HP resin were higher compared to IRA 402 (Cl^−^) resin for all concentrations studied.

### 3.4. Effect of Desorption Agent

**Figure 4:**
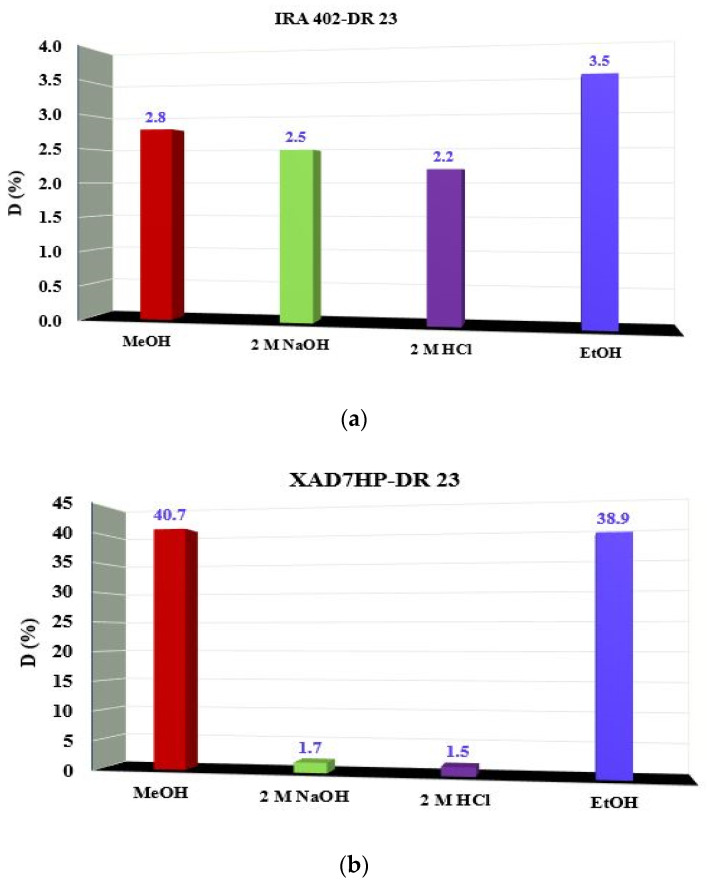
Effect of desorption agents for (**a**) IRA402-DR 23 and (**b**) XAD7HP-DR 23.

In order to verify if the resins obtained can be used for M^2+^ removal, the stability of chelate resins was evaluated. It is known that the majority of aqueous matrices have acidic pH. Chelated resin IRA 402-DR 23 keeps its form at 2 M (HCl and NaOH) and also in the presence of some common organic solvents (MeOH and EtOH); see Figure 4a. Therefore, if the interaction between the chelate agent and resin structure IRA 402 (Cl^−^) is governed by an ion exchange mechanism, the strategy follows the following hypothesis: Cl^−^ ions from 2 M HCl and HO^−^ from 2 M NaOH solutions tend to replace the anionic form of DR 23 through an ion exchange mechanism.

The high stability of the chelated resin is a consequence of physical adsorption that act together with ion exchange adsorption. For XAD7HP-DR 23 resin, the chelating agent remains loaded in the resin mass when the acidic (2M HCl) and basic (2M NaOH) solution is added. The influence of the desorption agent is observed for XAD7HP-DR 23 when 40.7% and 38.9% of the loaded chelating agent were released in the MeOH and EtOH (Figure 4b).

The XAD7HP-DR 23 is easy to prepare and the original resin XAD7HP can be recovered and used for obtaining chelating resin in a new study. The behavior of IRA 402-DR 23 in the organic eluent is insignificant and the chelating agent is not released. Similar results were obtained by the authors in a previous study [18,19,25].

### 3.5. Adsorption Kinetics

**Figure 5:**
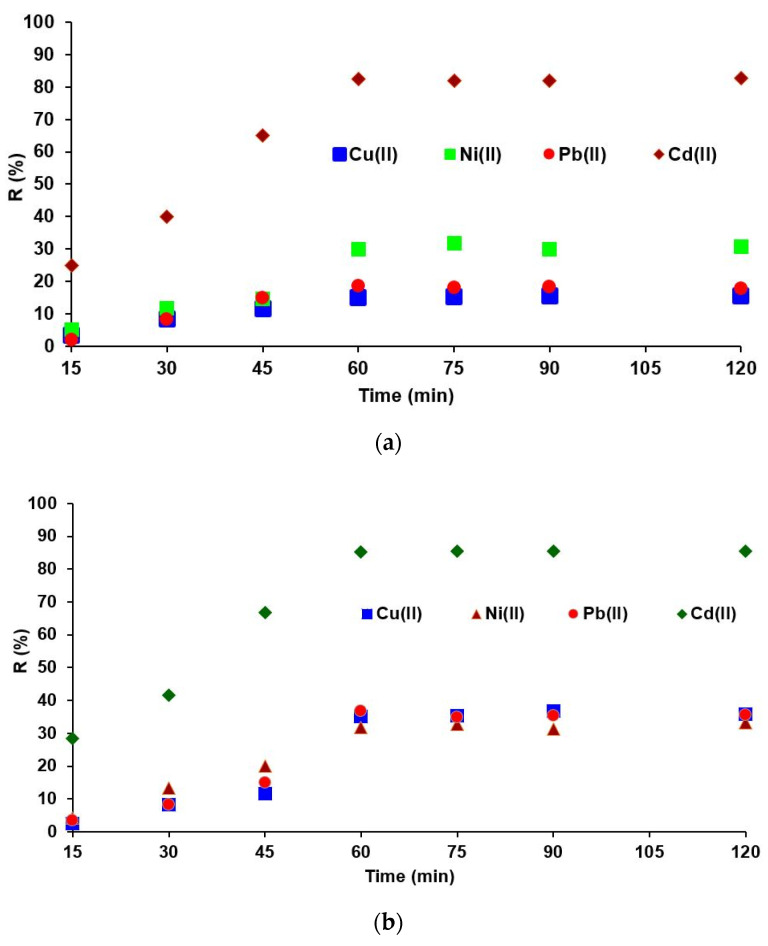
Removal of M^2+^ as a function of contact time on (**a**) IRA 402-DR23 and (**b**) XAD7HP-DR23.

The kinetics of the adsorption process necessary to reach the equilibrium between the liquid–solid phases (solutions of M^2+^- chelating resins) was studied ranging from 15 to 120 min. The experimental results obtained on the influence of the contact time (M^2+^- chelating resins) are presented in Figure 5. One result is that, from 60 to 120 min, the M^2+^adsorption on both chelating resins becomes slow and the percentage of M^2+^ retained varies insignificantly (see Figure 5a,b). In the next experiment, M^2+^ adsorption on both chelating resins, the optimal contact time was set to 60 min.

Experimental data obtained on the influence of contact time (interaction between M^2+^- chelating resin) were fitted by the pseudo-first- (Lagergren) and pseudo-second-order (Ho and McKay) kinetic models.

The pseudo-first- and -second-order kinetic models are described by Equations (5) and (6) [19,26]:

(5) $$\log\left(Q_{e}{-Q}_{t}\right){=\mathrm{logQ}}_{e}-\left(\frac{k_{1}}{2.303}\right)t$$

(6) $$\frac{t}{Q_{t}}=\frac{1}{k_{2\;}qQ_{e}2\;}+\frac{t}{Q_{e}}$$

where *k*_1_ (min^−1^) is the rate constant of the pseudo-first-order model; *Q_e_* and *Q_t_*a re the amount of the M^2+^ retained at equilibrium (*e*) of IRA 402-DR 23 and XAD7HP-DR 23 and at time (*t*) on IRA 402-DR 23 and XAD7HP-DR 23, respectively; and *k*_2_ (g/(mg·min)) is the rate constant of the pseudo-second-order kinetic model. From the graphical representation of *log*(*Q_e_* – *Q_t_*) vs. *t*, the constant *k*_1_ (min^−1^) and the amount retained at equilibrium (*Q_e_* calc. (mg/g)) were obtained.

Additionally, from the representation of *t*/*Q_t_* vs. *t,* the *k*_2_ constant and *Q_e_* calc. (mg/g) values were determined. Comparing the R^2^ results obtained for each metal ion when the pseudo-first- and -second-order kinetic model was fitted, it can be seen that the best values were obtained for the first kinetic model (Table 1).

### 3.6. Application of Chelating Resin toward M^2+^ Removal

In this work, the presence of functional groups existing in the structure of the chelating agent act with good affinity for M^2+^, binding the metal through a chelation or complexation mechanism.

The adsorption mechanism between chelating resins (IRA 402-DR 23 contained 21.2 mg DR 23/g of IRA 402 and XAD7HP-DR 23 contained 27.2 mg DR 23/g of XAD7HP) and M^2+^ can be explained by taking into consideration the following hypothesis: the DR 23 contains two azo groups (–N=N–) that can have a good affinity for M^2+^ (i) when azo groups can donate a pair of electrons chelating or complexing the M^2+^; (ii) by diffusion in the porous structure of chelating resins; (iii) by ion exchange between both sulfonate groups (SO_3_^−^) that are not employed employ in the first step when chelating resins were obtained; and (iv) by carbonyl groups (–C=O) of XAD7HP resin, which can be involved in a complexation mechanism by oxygen atoms of the –C=O group and the metal ions.

In order to check the applicability of the chelating resins, the batch mode was employed for selectivity M^2+^ adsorption. Therefore, the practical applicability of the modified resins was tested by seven synthetic mixtures of Cd^2+^, Cu^2+^, Ni^2+,^ and Pb^2+^ with 0.05 mg/L (S1), 0.1 mg/L (S2), 0.15 mg/L (S3), 0.2 mg/L (S4), 0.3 mg/L (S5), 0.4 mg/L (S6) and 0.6 mg/L (S7), respectively. As one can observe, R (%) values are greater for S1, S2 and S3 at low concentrations of M^2+^ (i.e., 0.05–0.15 mg/L). Subsequently, R (%) decreases with increasing concentration of M^2+^ (i.e., 0.2–0.6 mg/L) corresponding to S4, S5, S6 and S7.

The chelating resins showed a huge selectivity for Cd^2+^ even when 0.6 mg/L M^2+^ was used, where 85% and 83% were removed by the XAD7HP-DR 23 and IRA 402-DR 23, respectively.

**Figure 6:**
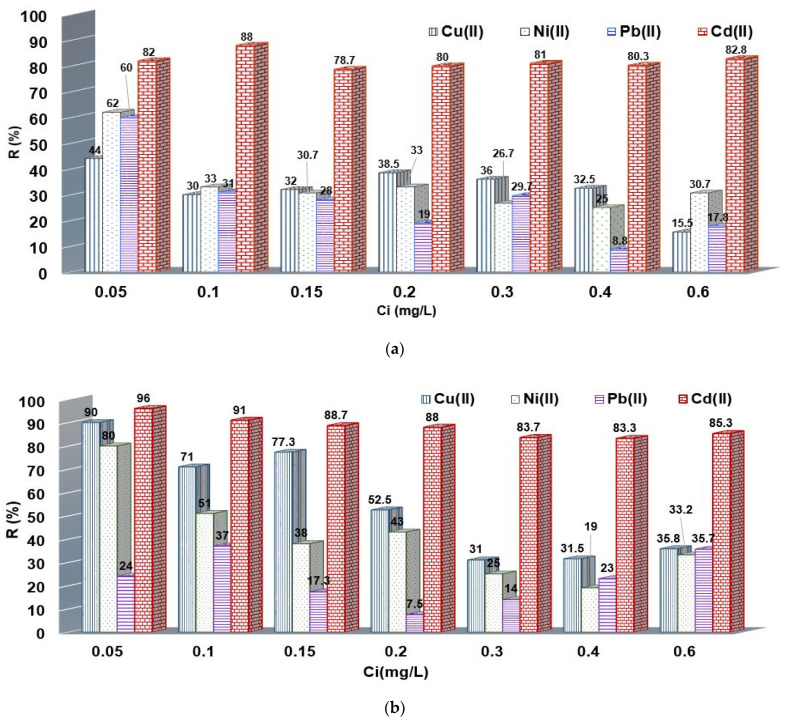
Cd^2+^, Cu^2+^, Ni^2+^ and Pb^2+^ removal on (**a**) IRA 402-DR 23 and (**b**) XAD7HP-DR 23.

For all cases, the M^2+^ efficiency on the XAD7HP-DR 23 was better highlighted compared with IRA 402-DR 23, giving promising results (see Figure 6a,b). On the other hand, Pb^2+^ was better removed by the IRA 402-DR 23 resin, and the R (%) was found to be 60% for S1, 31% for S2, 28% for S3, 19% for S4 and 30% for S5. In comparison, the R (%) of the XAD7HP-DR 23 for Pb^2+^ (S1, S3–S5) was under IRA 402-DR 23, except for S6 and S7, when XAD7HP-DR 23 removed double the amount of Pb^2+^.

Thus, IRA 402-DR 23 and XAD7HP-DR 23 provide a huge selectivity for Cd^2+^, while Cu^2+^ (0.73 Å), Ni^2+^ (0.69 Å) and Pb^2+^ (1.19 Å) were removed as a function of the ionic radius if taking into consideration the hypothesis presented in the previous study [24].

Simultaneously, M^2+^ removal on the chelating resin is presented in Figure 6a,b.

The total adsorption capacity (Q_t_) for the highest M^2+^ concentration tested (0.6 mg/L) was 0.0718 mg M^2+^/g of IRA 402-DR 23 and 0.1140 mg M^2+^/g of XAD7HP-DR 23. Therefore, it was determined that the Q_t_ of removed M^2+^ did not exceed the adsorption capacity of the chelating resins tested.

Additionally, a literature overview of other adsorbents used for the removal of harmful metal ions was studied (Table 2).

Analyzing the data presented in Table 2 shows that a smaller adsorption capacity can be observed for IRA 402-DR 23 and for XAD7HP-DR 23 resin for M^2+^ removal if we refer to other resins presented comparatively. It is mandatory to mention that these results were obtained in strongly different conditions. For instance, in [29], the initial concentration was 200 mg/L, in [30] it was 3000 mg/L, in [31] it was 322 mg/L, and in [32] it was 100 mg/L. Additionally, it is worth mentioning that most of these heavy metals are in the ppt-ppb level. Considering these data, even if the IRA 402-DR 23 and XAD7HP-DR 23 resins have a moderately smaller adsorption capacity for M^2+^ adsorption, they could be a solution for a real aqueous sample with concentrations in the range of ppt-ppm; perhaps these systems can be used in association with those presented in [29,30,31,32] as a final step in heavy metal removal.

### 3.7. Adsorption Isotherms

The interaction between divalent metal ions and chelated resins was studied based on the theoretical isotherm models of Langmuir (Equations (7)–(8)), Freundlich (Equation (9)), and Dubinin Radushkevich (Equations (10)–(12)) [19].

(7) $$\frac{C_{e}}{Q_{e}}=\frac{1}{{\mathrm{bQ}}_{0}}+\frac{C_{e}}{Q_{0}}$$

(8) $$R_{L}=\frac{1}{1+bC_{0}}$$

(9) $${\mathrm{lnQ}}_{e\;}{=\mathrm{lnK}}_{f}+\frac{1}{n}{\mathrm{lnC}}_{e}$$

(10) $$\ln Q_{e}=\ln q_{m}-\beta \varepsilon^{2}\;$$

(11) $$\varepsilon =\mathrm{RTln}(1+\frac{1}{C_{e}})$$

(12) $$E=\frac{1}{\sqrt{2\beta }}$$

where *Q*_0_ is the maximum adsorption of IRA 402-DR 23 and XAD7HP-DR 23; *b* is the Langmuir constant correlated with the adsorption capacity of IRA 402-DR 23 and XAD7HP-DR 23; *C*_0_ (mg/L) is the highest initial concentration of M^2+^ adsorbed by the chelating resin at equilibrium; and *R_L_* is the separation factor. The value of *R_L_* shows whether the adsorption process is favorable 0 < *R_L_* < 1, unfavorable *R_L_* > 1, linear *R_L_* = 1, or irreversible *R_L_* = 0. *K_f_* is the Freundlich constant, *n* is the constant correlated with the energetic heterogeneity of the adsorption sites; *q_m_* is the adsorption capacity of a theoretical monolayer (mg/g); *β* is the constant of adsorption energy (mol^2^/J^2^); *Ɛ* is the Polonyi potential; and *E* (KJ/mol) is the mean of the free energy.

The value of *R_L_* was evaluated by applying Equation (9) for all M^2+^ concentrations studied (i.e., 0.05–0.6 mg/L). As one can observe from Table 3, the adsorption process was determined to be favorable (0 < *R_L_* < 1). Linear regression isotherms were applied to better understand the adsorption mechanism for the removal of the M^2+^.

The applicability of theoretic isotherm models was evaluated by taking into consideration the R^2^ value. Taking into account the high value of the R^2^, we can issue the theoretical hypothesis that Cd^2+^, Cu^2+^, and Ni^2+^ are retained on the heterogeneous surface of IRA 402-DR 23 described by the Freundlich model, while the Pb^2+^ is adsorbed by the homogeneous surface of IRA 402-DR 23 described by the Langmuir model.

Additionally, for the following chelating resin, Cu^2+^ is retained on the homogeneous surface of the XAD7HP-DR 23, described by the Langmuir model (Table 3). As one can see in Table 3, Cd^2+^, Pb^2+^ and Ni^2+^ are removed by the heterogeneous surface of the XAD7HP-DR 23 according to the Freundlich model.

Moreover, the E (KJ/mol) values obtained by applying the Dubinin Raduschevich model suggest that M^2+^ is retained by chemical adsorption on the IRA 402-DR 23 and XAD7HP-DR 23 (Table 3).

### 3.8. Effect of the Desorption Agent from the Chelating Resins Loaded with M^2+^

**Figure 7:**
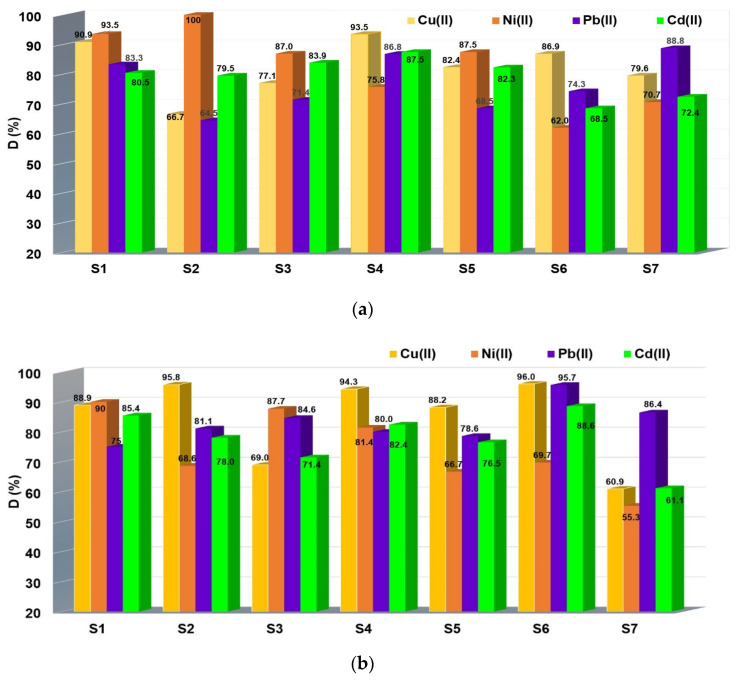
Effect of 2 M HCl solution on M^2+^ desorption from chelating material exhausted (**a**) IRA402-DR 23-M^2+^ and (**b**) XAD7HP-DR 23-M^2+^, where S1 to S7 represents the solid phases of IRA 402-DR 23-M^2+^ and XAD7HP-DR 23-M^2+^ obtained in Section 3.6. that were subjected to desorption studies.

Desorption studies of M^2+^ from chelated resins were also evaluated. In most applications, when the charged metal ions on the chelating resin exceed their ion-exchange capacity, the chelating resin is considered exhausted and must be regenerated. Thus, once the resin is exhausted, it no longer retains the metal ions from the solution, a fact that is monitored by continuous measurement of effluent. The exhausted resin is regenerated with solutions in which the ion concentration must be high enough to remove the metal ions adsorbed in the chelating resin mass subject to regeneration. For chelating resins, the desorption agent can be a mineral acid [33,34]. In our paper, after adsorption of Cd^2+^, Cu^2+^, Ni^2+^ and Pb^2+^ onto chelating resins, the release of those was evaluated using 2 M HCl. A high acid concentration was used, taking into consideration the greater stability of chelating resins presented in Section 2.9 and Section 3.4. The obtained results are presented in Figure 7a,b. It is observed that all M^2+^ retained on resins’ mass were found quantitatively in the supernatant solution of 2 M HCl. The M^2+^ desorption order from the IRA402-DR 23-M^2+^ was Ni^2+^ > Cu^2+^ > Cd^2+^ > Pb^2+^, while for XAD7HP-DR 23-M^2+^resin, the behavior Cu^2+^ > Pb^2+^ > Cd^2+^ > Ni^2+^ was obtained. Therefore, the desorption studies suggest that these resins can be reused, justifying the cost of production. A similar desorption experiment was related by Shy et al. when the dithiocarbamate chelating resin was evaluated from simulated wastewater Co^2+^. The desorption studies were performed using 0.1 M HCl when 99.1% Co^2+^ was released into the solution [35]. Chloromethylated polystyrene resin (PS-Cl) together with D380 (PS-NH_2_) were functionalized with citric acid for Fe^3+^, Al^3+^, Pb^2+,^ Cu^2+^, Cd^2+^ and Hg^2+^ removal. The obtained results confirm the hypothesis that, in a strongly acidic environment, the capacity of a combination of metal ions and resins would decrease due to the fact that the COO^−^ group would be non-ionized. Metal ions were desorbed using the following experimental conditions: 20 mL HCl and 24 h contact time between liquid and solid phases. The majority of the metal was desorbed at a ratio exceeding 98% in 0.5% HCl, except Fe^3+^, when only 74.6% was detected. When the acidity of the eluent was increased to 3.0% HCl, Fe^3+^ was efficiently desorbed by more than 98%. Considering the results obtained, modified resins with citric acid can be easily regenerated with 0.5% or 3.0% HCl [36].

### 3.9. FTIR Evaluation of the Chelating Resin–Metal Interaction

**Figure 8:**
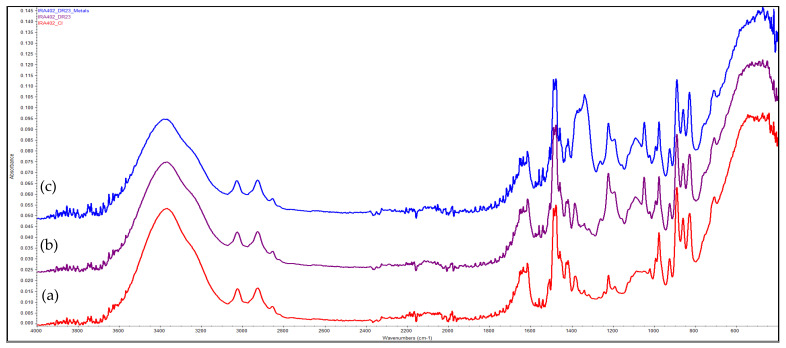
FTIR spectra of (**a**) IRA 402 (Cl^−^), (**b**) IRA 402-DR23 and (**c**) IRA 402-DR23-M^2+^.

**Figure 9:**
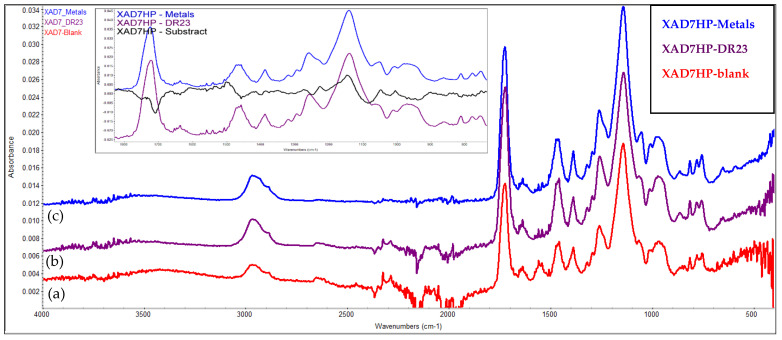
FTIR spectra of (**a**) XAD7HP, (**b**) XAD7HP-DR23 and (**c**) XAD7HP-DR23-M^2+^; insert represents the subtraction of the FTIR spectra of XAD7HP-DR23 and XAD7HP-DR23-M^2+^.

FTIR can be used in the evaluation of the interaction between the chelating resin and the metal ions [37]. Therefore, two chelating resins (IRA 402-DR23 and XAD7HP-DR 23) were obtained starting from Amberlite IRA 402 (Cl^−^) and Amberlite XAD7HP produced by the same supplier (Sigma Aldrich) and tested comparatively for M^2+^ removal. Based on the FTIR data, we can observe that absorption of metals leads to a strong modification of the FTIR spectra when IRA402-DR23 adsorbs metal cations, and consequently, a strong peak centered at 1338.52 cm^−1^ is observed (over which some smaller peaks centered at 1362.21 and 1374.06 cm^−1^ are overlapping) (Figure 8). Additionally, the intensity of the peaks involved in these interactions changes their intensity. In the case of the XAD7HP series (Figure 8), the presence of some specific bands cannot be identified, and the relative intensity of the peaks is also not visibly changing. Consequently, the subtraction of the FTIR peaks of the XAD7HP-DR23-Metals and XAD7HP-DR23 was performed. The most important changes occur in the 900–1800 cm^−1^ region, and thus, this area is presented as an insert in Figure 9. Based on the subtracted spectrum, it can be seen that more peaks of the XAD7HP-DR23 (1722.88, 1457.61, 1257.22 or 1139.61) are modified. Based on these changes, it can be concluded with no doubt that interactions develop between the two supports modified with DR23 and the metal cations.

### 3.10. SEM Analysis

**Figure 10:**
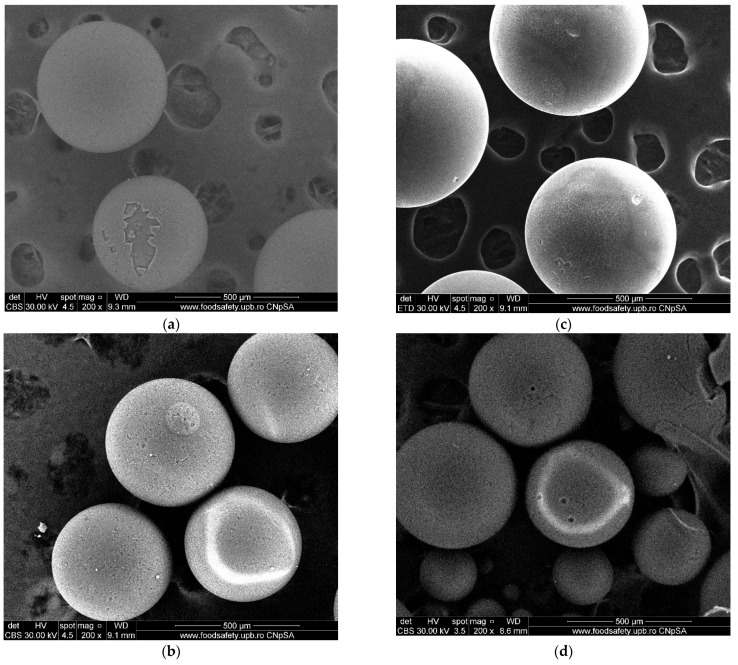
SEM Images of (**a**) IRA 402-DR23, (**b**) IRA 402-DR23-M^2+^, (**c**) XAD7HP-DR23 and (**d**) XAD7HP-DR23-M^2+^.

SEM images of the IRA 402-DR 23, IRA 402-DR 23-M^2+^, XAD7HP-DR 23 and XAD7HP-DR 23-M^2+^ are shown in Figure 10a–d. For this aim, different beds of chelated resins before and after M^2+^ interaction were selected. First, it can be observed that the absorption of the metal cations did not change the size of the DR23-modified microbeads (IRA402-DR23 or XAD7HP-DR23); thus, it can shown that no significant degradation occurred during the absorption process. At the surface, some minor changes appeared as a consequence of the adsorption. For instance, in the case of IRA 402-DR23/IRA 402-DR23-M^2+^ (Figure 10a,c), the surface became rougher, while, in the case of XAD7HP-DR23/XAD7HP-DR23-M^2+^ (Figure 10b,d), the density of the surface pores decreases.

Results of the metal removal of chelating resin were quantified through SEM analysis. Thus, Fatah et al. presented SEM analysis of La^3+^ adsorption onto functionalized Amberlite XAD-4 with Cyanex 921 when the metal ions were coordinated by the oxygen atoms of the chelating agent. The presence of La^3+^ onto walls’ spheres by SEM images was highlighted [38]. Additionally, chelating resin H^+^ Dowex-M4195 was tested for Cu^2+^ adsorption. The SEM image of chelating resin loaded with Cu^2+^ has a smoother surface reported with the resin used before adsorption [39].

## 4. Conclusions

In summary, new chelated resins, i.e., IRA 402-DR 23 and XAD7HP-DR 23, were obtained, and their applicability for Cd^2+^, Cu^2+^, Ni^2+^ and Pb^2+^ removal was studied. It was determined that the chelating procedure of IRA 402 (Cl^−^) and XAD7HP improves with the interaction time and the initial concentration of the DR 23, while the pH effect is insignificant.

Considering the excellent stability of the IRA 402-DR 23 and XAD7HP-DR 23 in an acidic medium, 2 M HCl can be used as an eluent for M^2+^ removed from chelating resins when the form of the resins does not need to be modified.

Additionally, if the MeOH or EtOH is used, the possibility of XAD7HP-DR 23 resin regeneration increases, allowing the initial XAD7HP resin to be recovered. At the same time, the overlap of the two mechanisms that contribute to the IRA 402-DR 23 resin gives high stability in a strongly acidic, basic, and also organic environments. The lower adsorption capacity obtained for XAD7HP (27.12 mg/g) and for IRA 402 (Cl^−^) (21.2 mg/g) results in the inability of DR 23 to access the internal structure of the resins.

Therefore, according to our study, the use of acrylic ester resin (XAD7HP) is better indicated when using a chelating agent with a high molecular structure. The low adsorption capacity determined experimentally compared with the ion exchange capacity of the IRA 402 (Cl^−^) resin showed that only a small part of the quaternary amino groups were involved in the DR 23 adsorption through an ion exchange mechanism predominating the π–π interactions between the styrene–divinyl benzene structure of the resin and the aromatic ring of the chelating agent.

The chelating resins were successfully applied for simultaneous M^2+^ removal from acid samples using the AAS method. The experimental data are best fitted by the pseudo-first-order kinetic model, which suggests that the adsorption rate is dependent on the adsorption capacity of the chelated resins in environmentally relevant concentrations (0.05–0.6 mg/L).

For both chelated resins, the value of R_L_ obeys the rule that 0 < R_L_ < 1, which suggests that the adsorption process of M^2+^ is favorable. Additionally, the M^2+^ adsorption on chelating resins is confirmed by the n values being with n > 1 in the Freundlich model.

Using 2 M HCl, efficient desorption of M^2+^ from chelating resins was obtained, such that the obtained were also recyclable.

Consequently, both chelating materials exhibited the highest selectivity for Cd^2+^, followed by Cu^2+^, Ni^2+^ and Pb^2+^, from water polluted with acid.

## Figures and Tables

**Table 1:** Kinetic constants of M^2+^ adsorption on the IRA 402-DR 23 and XAD7HP-DR 23.

Chelating Resins	IRA402-DR 23				XAD7HP-DR 23			
Kinetic Models	Cd^2+^	Cu^2+^	Ni^2+^	Pb^2+^	Cd^2+^	Cu^2+^	Ni^2+^	Pb^2+^
Pseudo-first-order model								
k_1_ (min^−1^)	0.040	0.058	0.023	0.046	0.060	0.026	0.050	0.057
Q_e_ calc. (mg/g)	21.7	49.3	28.0	70.1	10.3	31.2	25.0	18.3
* Q_e_ exp. (mg/g)	0.49	0.009	0.019	0.011	0.050	0.024	0.020	0.022
R^2^	0.950	0.953	0.700	0.900	0.920	0.958	0.953	0.861
Pseudo-second-order								
k_2_ (g/(mg∙min))	0.240	0.230	0.005	0.004	0.014	0.010	0.050	0.048
Q_e_ calc (mg/g)	0.006	0.078	0.150	0.170	0.340	0.30	0.096	0.06
R^2^	0.886	0.886	0.200	0.100	0.908	0.910	0.250	0.201

* Values obtained for the influence of contact time, using experimental conditions: contact time 60 min, 0.6 mg/L M^2+^ and 0.5 g of each chelating resin, shaken at 175 rpm 25 ± 2 °C.

**Table 2:** Comparison of IRA 402-DR 23 and XAD7HP-DR 23 with other adsorbents used for the removal of hazardous metal ions.

Matrices	Chelating Agents	Adsorption Capacities (mg/g)								Ref.
		Cd^2+^	Co^2+^	Cr^3+^	Cu^2+^	Mn^2+^	Ni^2+^	Pb^2+^	Zn^2+^	
Amberlite IRA 402 (Cl^−^)	DR 23	0.050	-	-	0.009	-	0.0018	0.011	-	This study
Amberlite XAD7HP	DR 23	0.051	-	-	0.022	-	0.020	0.021	-	This study
Poly(diacetonitrile methacrylamide-coinylimidazole)	Polyvinyl- imidazole	29.3	31.6	29.3	27.3	35.5	31.7	39.8	32.3	[29]
Glycidyl methacrylate	N,N-methylene biscarylamid	369	73	40.6	151	94.1	99.2	136.8	92.2	[30]
Amberlite XAD4	1-(2-thiazolylazo) -2-naphthol		- -	-	8	-	-	-	-	[31]
Amberlite XAD2	Calcein blue	-	-	-	27	-	-	--		[32]

**Table 3:** Langmuir, Freundlich and Dubinin Radushkevich isotherm constants regarding M^2+^ adsorption on chelated resins.

Chelating Resins	IRA402-DR 23				XAD7HP-DR 23			
Isotherm Models	Cd^2+^	Cu^2+^	Ni^2+^	Pb^2+^	Cd^2+^	Cu^2+^	Ni^2+^	Pb^2+^
Langmuir								
Q_0_ (mg/g)	0.324	0.013	0.029	0.008	0.069	0.018	0.015	0.168
b (L/mg)	1.478	7.732	2.296	9.701	16.813	17.043	9.238	0.122
R^2^	0.160	0.775	0.401	0.455	0.674	0.754	0.555	0.204
R_L_	0.576	0.582	0.703	0.491	0.104	0.214	0.352	0.974
Freundlich								
K_F_	2.429	1.879	1.746	1.363	1.821	1.310	1.379	2.365
1/n	0.888	0.631	0.558	0.310	0.600	0.270	1.321	0.861
n	1.127	1.586	1.794	3.230	1.668	3.704	3.112	1.162
R^2^	0.942	0.792	0.830	0.421	0.968	0.732	0.636	0.537
Dubinin–Radushkevich								
q_m_ (mg/g)	13.448	70.0	85.151	152.0	19.101	66.622	94.332	100.0
β (mol^2^/kJ^2^)	0.2 × 10^−7^	0.3 × 10^−7^	0.2 × 10^−7^	0.1 × 10^−7^	0.1 × 10^−7^	0.7 × 10^−8^	0.9 × 10^−8^	0.3 × 10^−7^
E (KJ/mol)	5000	4082	5000	7071	7071	8452	7454	4083
R^2^	0.914	0.838	0.656	0.344	0.906	0.7060	0.515	0.445

## Data Availability

The data supporting the reported results are available on request from the authors.

## References

[1] Ahmad A., Siddique J.A., Laskar M.A., Kumar R., Mohd-Setapar S.H., Khatoon A., Shiekh R.A. (2015). New generation Amberlite XAD resin for the removal of metal ions: A review. J. Environ. Sci..

[2] Baby R., Hussein M.Z., Abdullah A.H., Zainal Z. (2022). Nanomaterials for the treatment of heavy metal contaminated water. Polymers.

[3] Balali-Mood M., Naseri K., Tahergorabi Z., Khazdair M.R., Sadeghi M. (2021). Toxic mechanisms of five heavy metals: Mercury, lead, chromium, cadmium, and arsenic. Front. Pharmacol..

[4] Popescu F., Trumić M., Cioabla A.E., Vujić B., Stoica V., Trumić M., Opris C., Bogdanović G., Trif-Tordai G. (2022). Analysis of Surface Water Quality and Sediments Content on Danube Basin in Djerdap-Iron Gate Protected Areas. Water.

[5] Chu S., Feng X., Liu C., Wu H., Liu X. (2022). Advances in Chelating Resins for Adsorption of Heavy Metal Ions. Ind. Eng. Chem. Res..

[6] Zafar M.N., Aslam I., Nadeem R., Munir S., Rana U.A., Khan S.U.-D. (2015). Characterization of chemically modified biosorbents from rice bran for biosorption of Ni(II). J. Taiwan Inst. Chem. Eng..

[7] Nadeem R., Zafar M.N., Afzal A., Hanif M.A., Saeed R. (2014). Potential of NaOH pretreated *Mangifera indica* waste biomass for the mitigation of Ni(II) and Co(II) from aqueous solutions. J. Taiwan Inst. Chem. Eng..

[8] Zafar M.N., Parveen A., Nadeem R. (2013). A pretreated green biosorbent based on Neem leaves biomass for the removal of lead from wastewater. Desalination Water Treat..

[9] Zhang X., Ma J., Zou B., Ran L., Zhu L., Zhang H., Ye Z., Zhou L. (2022). Synthesis of a novel bis Schiff base chelating resin for adsorption of heavy metal ions and catalytic reduction of 4-NP. React. Funct. Polym..

[10] Suwannahong K., Sripirom J., Sirilamduan C., Thathong V., Kreetachart T., Panmuang P., Deepatana A., Punbut S., Wongcharee S. (2022). Selective Chelating Resin for Copper Removal and Recovery in Aqueous Acidic Solution Generated from Synthetic Copper-Citrate Complexes from Bioleaching of E-waste. Adsorp. Sci. Technol..

[11] Benettayeb A., Ghosh S., Usman M., Seihoub F.Z., Sohoo I., Chia C.H., Sillanpää M. (2022). Some Well-Known Alginate and Chitosan Modifications Used in Adsorption: A Review. Water.

[12] Elbadawy H.A., Abdel-Salam A.H., Khalil T.E. (2021). The impact of an Amberlite XAD-16-based chelating resin for the removal of aqueous Cd(II) and Pb(II) ions. Microchem. J..

[13] Kocaoba S. (2022). Determination of some heavy metals from aqueous solutions using modified Amberlite XAD-4 resin by selective solid-phase extraction. J. Anal. Sci. Technol..

[14] Cai Y., Wang M., Zeng Y., Hu B., Wang Y., Yuan L., Feng W. (2022). Efficient and selective lanthanide recovery from highly acidic solutions by using a porous pillar[5]arene-based diglycolamide impregnated resin. Hydrometallurgy.

[15] Van Nguyen N., Lee J.-C., Jeong J., Pandey B. (2013). Enhancing the adsorption of chromium(VI) from the acidic chloride media using solvent impregnated resin (SIR). Chem. Eng. J..

[16] Benmansour Y., Didi M.A., Abderarhim O. (2022). Amberlite IRA-93 and modified Amberlite IRA-93 resins for the uranyl ions extraction: Optimization through factorial design methodology. Desalination Water Treat..

[17] Sharaf M., Yoshida W., Kubota F., Goto M. (2019). A novel binary-extractant-impregnated resin for selective recovery of scandium. J. Chem. Eng. Jpn..

[18] Marin N.M. (2022). Natural and Synthetic Polymers Modified with Acid Blue 113 for Removal of Cr^3+^, Zn^2+^ and Mn^2+^. Polymers.

[19] Marin N.M., Stanculescu I. (2022). Removal of procainamide and lidocaine on Amberlite XAD7HP resin and of As(V), Pb(II) and Cd(II) on the impregnated resin for water treatment. Mater. Chem. Phys..

[20] Ghanbarizadeh P., Parivazh M.M., Abbasi M., Osfouri S., Dianat M.J., Rostami A., Dibaj M., Akrami M. (2022). Performance Enhancement of Specific Adsorbents for Hardness Reduction of Drinking Water and Groundwater. Water.

[21] Alswieleh A.M. (2022). Efficient Removal of Dyes from Aqueous Solution by Adsorption on L-Arginine-Modified Mesoporous Silica Nanoparticles. Processes.

[22] Mosoarca G., Popa S., Vancea C., Dan M., Boran S. (2022). Removal of Methylene Blue from Aqueous Solutions Using a New Natural Lignocellulosic Adsorbent—Raspberry (*Rubus idaeus*) Leaves Powder. Polymers.

[23] Kali A., Amar A., Loulidi I., Hadey C., Jabri M., Alrashdi A.A., Lgaz H., Sadoq M., El-kordy A., Boukhlifi F. (2022). Efficient Adsorption Removal of an Anionic Azo Dye by Lignocellulosic Waste Material and Sludge Recycling into Combustible Briquettes. Colloids Interfaces.

[24] Marin N.M. (2022). Maize Stalk Obtained after Acid Treatment and Its Use for Simultaneous Removal of Cu^2+^, Pb^2^+, Ni^2+^, Cd^2+^, Cr^3+^ and Fe^3+^. Polymers.

[25] Marin N.M., Stanculescu I. (2021). Application of amberlite IRA 402 resin adsorption and laccase treatment for acid blue 113 removal from aqueous media. Polymers.

[26] Marin N.M., Pascu L.F., Demba A., Nita-Lazar M., Badea I.A., Aboul-Enein H. (2019). Removal of the Acid Orange 10 by ion exchange and microbiological methods. Int. J. Environ. Sci. Technol..

[27] Awasthi A., Datta D. (2019). Application of Amberlite XAD-7HP resin impregnated with Aliquat 336 for the removal of Reactive Blue-13 dye: Batch and fixed-bed column studies. J. Environ. Chem. Eng..

[28] Ion Exchange Capacity. http://dardel.info/IX/capacity.html.

[29] Yavuz E., Tokalıoğlu Ş., Erkılıç H., Soykan C. (2017). Novel chelating resin for solid-phase extraction of metals in certified reference materials and waters. Anal. Lett..

[30] Jiang J., Ma X.-S., Xu L.-Y., Wang L.-H., Liu G.-Y., Xu Q.-F., Lu J.-M., Zhang Y. (2015). Applications of chelating resin for heavy metal removal from wastewater. e-Polymers.

[31] Goodwin W.E., Rao R.R., Chatt A. (2013). Reversed-phase extraction chromatography-neutron activation analysis (RPEC-NAA) for copper in natural waters using Amberlite XAD-4 resin coated with 1-(2-thiazolylazo)-2-naphthol. J. Radioanal. Nucl. Chem..

[32] Moniri E., Ahmad Panahi H., Karimi M., Ahmad Rajabi N., Faridi M., Manoochehri M. (2011). Modification and characterization of amberlite XAD-2 with calcein blue for preconcentration and determination of copper(II) from environmental samples by atomic absorption spectroscopy. Korean J. Chem. Eng..

[33] Allam E.M., Lashen T.A., Abou El-Enein S.A., Hassanin M.A., Sakr A.K., Hanfi M.Y., Sayyed M., Al-Otaibi J.S., Cheira M.F. (2022). Cetylpyridinium Bromide/Polyvinyl Chloride for Substantially Efficient Capture of Rare Earth Elements from Chloride Solution. Polymers.

[34] Ibrahium H., Abdel Aal M., Awwad N., Atia B., Ali H., Gado M., Hakami R., Cheira M. (2022). Solid-liquid separation of V(V) from aqueous medium by 3-(2-hydroxy phenyl)-imino-1-phenyl butan-1-one Schiff base immobilized XAD-2 resin. Int. J. Environ. Sci. Technol..

[35] Shi X., Fu L., Wu Y., Zhao H., Zhao S., Xu S. (2017). Functionalized dithiocarbamate chelating resin for the removal of Co^2+^ from simulated wastewater. Appl. Water Sci..

[36] Liu X., Xu L., Liu Y., Zhou W. (2018). Synthesis of citric acid-modified resins and their adsorption properties towards metal ions. R. Soc. Open Sci..

[37] Simonescu C.M., Lavric V., Musina A., Antonescu O.M., Culita D.C., Marinescu V., Tardei C., Oprea O., Pandele A.M. (2020). Experimental and modeling of cadmium ions removal by chelating resins. J. Mol. Liq..

[38] Fatah A., Elashry S.M. (2022). La(III) Separation by Tri Octyl Phosphine Oxide (Cyanex 921) Based on Amberlite Xad-4 Chelating Resin. J. Inorg. Organomet. Polym..

[39] Suwannahong K., Sirilamduan C., Deepatana A., Kreetachat T., Wongcharee S. (2022). Characterization and Optimization of Polymeric Bispicolamine Chelating Resin: Performance Evaluation via RSM Using Copper in Acid Liquors as a Model Substrate through Ion Exchange Method. Molecules.

